# Affiliate performance feedback and technology innovation: The mediating effect of the parent’s response

**DOI:** 10.3389/fpsyg.2022.1056867

**Published:** 2023-01-25

**Authors:** Li Zheng, Binbin Sun

**Affiliations:** Business School, Qingdao University, Qingdao, China

**Keywords:** performance feedback, parent functions, technology innovation, hierarchy management, resource allocation

## Abstract

The relationship between firm performance feedback and technology innovation has been studied extensively, but limited attention has been paid to factors that mediate this relationship. We examine how a parent firm addresses an affiliate’s performance level and its influence on the affiliate’s technology innovation. Integrating the behavioral theory of the firm and the concept of parent functions, we argue that the parent firm addresses the affiliate’s performance level in two ways: hierarchy management and resource allocation. Specifically, unlike the case of outperforming affiliates, the parent firm facilitates the technology innovation of underperforming affiliates through hierarchy management and resource allocation. Regression analyses of 2010–2020 data of listed affiliates belonging to Chinese business groups provide strong evidence supporting our conjecture. Our study sheds light on the importance of considering the parent’s influence when affiliates adopt technology innovation in light of its performance feedback.

## Introduction

Based on the behavioral theory of the firm (BTOF; Cyert and March, 1963; Argote and Greve, 2007), performance feedback has a significant influence on a firm’s technology innovation (Greve, 2003; Chen and Miller, 2007; Bromiley and Washburn, 2011; Ye et al., 2021). However, unlike stand-alone firms, affiliated firms need to consider the influence of their parent firm (Vissa et al., 2010; Gaba and Joseph, 2013; Joseph et al., 2016; Sengul and Obloj, 2017; Dutt and Joseph, 2019; Rhee et al., 2019). An affiliate’s performance level impacts the business group’s welfare, and the parent firm, as the ultimate shareholder, is motivated to guide the affiliate’s technology innovation (Vissa et al., 2010; Rhee et al., 2019). Prior studies have focused mainly on the direct correlation between performance feedback and technology innovation (Greve, 2003; Chen and Miller, 2007; Bromiley and Washburn, 2011; Ye et al., 2021), devoting limited attention to how the parent firm addresses the affiliate’s performance level and how this contributes to the affiliate’s technology innovation.

Moreover, exactly how parent functions (i.e., hierarchy management and resource allocation) influence the affiliate’s technology innovation is not sufficiently clear. Some scholars argued that affiliates can improve their innovative capabilities by conforming with their parent firm’s strategies (Hu et al., 2019; Zheng et al., 2022), while others contended that parent functions constrain the affiliate’s technology innovation (Asakawa et al., 2018; Wang and Wang, 2021). In essence, the debate is about how parent functions influence the affiliate’s strategic decisions. Drawing on the BTOF, we surmise that the affiliate’s performance level significantly impacts the business group’s welfare, and the parent firm tends to take measures to guide the affiliate’s decision-making (Sengul and Obloj, 2017; Sengul et al., 2019) to ensure alignment with the group’s aspirations. We focus particularly on two crucial parent functions: hierarchy management and resource allocation (Meyer et al., 2020), and examine the mediating effect of parent functions on the relationship between the affiliate’s performance feedback and technology innovation, to reveal how the parent firm’s approach to address the affiliate’s performance level impacts the affiliate’s technology innovation.

This paper contributes to the literature in several ways. Firstly, it offers a novel perspective on the research regarding the BTOF. Prior studies have shown that performance feedback significantly impacts technology innovation (Greve, 2003; Chen and Miller, 2007; Bromiley and Washburn, 2011; Ye et al., 2021), but the underlying mechanisms in this relationship remain underexplored. The gap limits our understanding of how performance feedback impacts technology innovation. In this study, we combine the BTOF and the concept of parent functions to clarify the influencing mechanisms of parent functions on the technology innovation of affiliates with different performance levels, thereby extending the BTOF research.

Secondly, we identify the ways a parent firm responds to its affiliate’s performance level. We focus on two parent functions: hierarchy management and resource allocation and find that the changes in parent functions are contingent upon the affiliate performance feedback. Our study contributes to the understanding of parent functions by providing a behavioral account on affiliate performance feedback.

Thirdly, we uncover the underlying mechanisms in the relationship between affiliate performance feedback and technology innovation. Our study depicts how the parent firm responds to the affiliate’s performance level and its influence on the affiliate’s technology innovation, thus advancing parent function research by clarifying the contingency factors that promote or impede affiliates’ technology innovation. We also provide some suggestions for the parent firm to guide its affiliates’ technology innovation.

## Theory and research background

### Affiliate performance feedback and parent firm’s attention

To investigate how a parent firm addresses an affiliate’s performance level, we apply the BTOF (Cyert and March, 1963; Argote and Greve, 2007). The core of the BTOF is that firms’ risk decisions are subject to the comparison between firm performance and its aspired level (Cyert and March, 1963; Greve, 2003). A firm sets its aspired performance level or target as a reference point for evaluating its performance, and treats it as the benchmark for whether firm performance is satisfactory (Cyert and March, 1963; Argote and Greve, 2007). If firm performance is above the aspired level (receiving positive performance feedback), the firm is satisfied with its returns and has less motivation to engage in risky decisions (Greve, 2003; Hoskisson et al., 2017). If firm performance is below the aspired level (receiving negative performance feedback), the firm’s routines are not well suited for the external market, which prompts the firm’s search for solutions (Cyert and March, 1963; Greve, 2003; Choi et al., 2019). In this scenario, some level of risk-taking is necessary for the firm to attain its aspired level (Hoskisson et al., 2017; Posen et al., 2018; Li and Vermeulen, 2021).

Attention refers to “the noticing, encoding, interpreting, and focusing of time and effort by organizational decision-makers” (Ocasio, 1997, p.189). Identifying the driving factors of attention distribution helps in exploring the influence of parent functions, as the parent firm guides affiliates’ strategic decisions according to its level of attention (Belenzon et al., 2019; Dutt and Joseph, 2019; Rhee et al., 2019; Bekmezci et al., 2022; Busenbark et al., 2022). Owing to bounded rationality (Simon, 1947), the attention capability is limited and the parent firm cannot similarly focus on all affiliates (Dutt and Joseph, 2019; Rhee et al., 2019; Eklund and Mannor, 2021). The performance level of the affiliate improves or damages the business group’s welfare (Jia et al., 2013; Joseph et al., 2016; Rhee et al., 2019), and the parent firm can distribute its attention among affiliates according to the affiliate’s performance level.

### A parent firm’s hierarchical management of its affiliates

The influence of the parent firm on its affiliates mainly refers to the managerial activities related to the formulation and implementation of affiliate strategies and operational activities that involve resource allocation and utilization to prompt the development of affiliates (Meyer et al., 2020). Many scholars have proposed that such activities for synergy management and resource sharing are the key functions of parent firms (Poppo, 2003; Belenzon et al., 2019). Considering this proposition, we conjecture that parent functions are manifested in two ways: hierarchy management and resource allocation.

Compared with affiliates, parent firms have greater prior knowledge and resources (Nell and Ambos, 2013; Zeng et al., 2018). To some extent, hierarchy management can prompt affiliates’ technology innovation (Ciabuschi et al., 2011; Palmié et al., 2016; Mahmood et al., 2017; Beugelsdijk and Jindra, 2018), as the parent firm provides some guidance and valuable resources to its affiliates (Raab et al., 2014; Zeng et al., 2018). However, hierarchy management is notably not always efficient. Some scholars have shown that parent firms lack sufficient knowledge and understanding of their affiliates’ activities owing to limited attention spans (Goold et al., 1998), and centralization leads to the affiliates’ limited development (Asakawa et al., 2018; Geleilate et al., 2020; Wang and Wang, 2021). Hence, how the parent firm manages its affiliates efficiently is a key issue.

### Resource allocation within the business group

Resource allocation among affiliates is one of the most essential functions of the parent firm (Belenzon et al., 2019; Sengul et al., 2019; Busenbark et al., 2022). The existing literature on resource allocation presents two views: winner picking and co-insurance. Winner picking theory holds that resource allocation is prioritized for outperforming affiliates to strengthen the core advantage (Williamson, 1975; Tan et al., 2018; Sengul et al., 2019; Lovallo et al., 2020), while co-insurance theory suggests that resource allocation is prioritized for underperforming affiliates to relieve operational risks (Jia et al., 2013; Busenbark et al., 2022). Regarding resource allocation, it is generally believed that outperforming affiliates that contribute more to the business group deserve more resources in return (Arrfelt et al., 2015). Why resources are not allocated in proportion to affiliates’ performance level is a puzzling question.

## Hypotheses development

### Affiliate performance feedback and hierarchy management

The affiliates’ performance level not only directly impacts the business group’s interest but also their responses to performance feedback affect the group development (Arrfelt et al., 2015; Sengul and Obloj, 2017). To ensure that affiliates’ strategic decisions are aligned with the business group’s aspiration, the parent firm is motivated to guide its affiliates’ decision-making through hierarchical management (Sengul and Obloj, 2017). Considering the advantages and disadvantages of hierarchy management (Raab et al., 2014; Palmié et al., 2016; Asakawa et al., 2018; Beugelsdijk and Jindra, 2018; Wang and Wang, 2021), we propose that the parent firm should adjust the hierarchy management according to the affiliate performance feedback and its possible responses.

The performance feedback of an affiliate affects the attention distribution of its parent firm. A performance level that is above the aspired level indicates that the affiliate has obtained a satisfactory return (Cyert and March, 1963; Argote and Greve, 2007). Simultaneously, the parent firm benefits from its ownership. As a response, it tends to pay less attention to such affiliates because the affiliate’s development is good (Sengul and Obloj, 2017). A negative performance feedback demonstrates that the affiliate has not realized its aspired level (Cyert and March, 1963; Argote and Greve, 2007), and the benefits for the business group decrease. In this scenario, the parent firm will pay more attention to such an affiliate to search for the problems and corresponding solutions (Meyer et al., 2020). Hence, compared with outperforming affiliates, underperforming affiliates attract more attention from the parent firm.

The hierarchy management depends on how the affiliates respond to the performance feedback. According to the BTOF, outperforming affiliates have a lower risk tolerance and tend to maintain the status quo (Hoskisson et al., 2017; Li and Vermeulen, 2021). The parent firm supports such affiliates’ decisions because of the satisfactory returns from these affiliates. The practice of strategic consistency allows the parent firm to appropriate a lower level of hierarchical management on these outperforming affiliates (Sengul and Obloj, 2017). Affiliates receiving a negative performance feedback have a higher risk tolerance and become more risk-seeking to achieve their aspired level (Hoskisson et al., 2017). Although risky decisions are critical for affiliates to improve their current situation, this may be accompanied by lower economic returns with a higher performance variance (Bowman, 1980; Henkel, 2009; Li and Vermeulen, 2021), which would further damage the business group’s welfare. Even if the parent firm makes repairs afterward, it cannot compensate for the loss caused by the affiliate’s decision-making (Sengul and Obloj, 2017). Hence, to guide the affiliate’s decision-making and maintain the group’s stable development, the parent firm would strengthen the hierarchy management on the underperforming affiliates.

Taken together, based on whether an affiliate realizes its aspired performance level and whether it engages in risky decisions, the parent firm decreases the level of hierarchy management on outperforming affiliates and increases that on underperforming affiliates.

*Hypothesis 1a*. A negative feedback on an affiliate’s performance is positively correlated with hierarchy management.

*Hypothesis 1b*. A positive feedback on an affiliate’s performance is negatively correlated with hierarchy management.

### Affiliate performance feedback and resource allocation

How to allocate resources within a business group is the parent firm’s responsibility, which covers all affiliates (Belenzon et al., 2019; Sengul et al., 2019; Busenbark et al., 2022). Owing to bounded rationality (Simon, 1947), the parent firm does not have sufficient time and knowledge to assess the value of its affiliates in all dimensions; thus, it focuses its attention on a limited set of variables such as performance (Arrfelt et al., 2013; Bardolet et al., 2017). Given that the affiliate’s performance level and its corresponding response impacts the business group’s welfare, the parent firm can influence the affiliate’s decision-making through resource allocation (Arrfelt et al., 2013; Jia et al., 2013; Dou et al., 2021). Hence, we propose that the resource allocation criteria depends on the affiliate performance feedback.

When an affiliate’s performance is above the aspired level, both the parent firm and affiliate are satisfied with the returns, and they tend to maintain the status quo (Hoskisson et al., 2017; Li and Vermeulen, 2021). Accordingly, it is not necessary for the parent firm to allocate resources to support the affiliate’s risky decisions. When an affiliate’s performance is below the aspired level, both the parent firm and affiliate become motivated to reach the aspired level through risky decisions. Considering the resource constraints of underperforming affiliates, the parent firm can allocate more resources to them (Arrfelt et al., 2013; Tan et al., 2018; Dou et al., 2021). Hence, the BTOF can shed light on why resource allocation deviates from the winner-picking approach based on the performance feedback and possible response of the affiliate.

Moreover, the internal transaction relationships among affiliates promote the response of resource allocation to the affiliate performance feedback (Jia et al., 2013). The business group’s competitive advantage is mainly reflected in knowledge sharing, technology transfer, and capital flow across the affiliates (Khanna and Yafeh, 2007; Hu et al., 2019), and the relationship between affiliates can become closer. Unlike the case of outperforming affiliates, the operating problems exposed by the underperforming affiliates may impede other affiliates’ development initiatives through the cooperation channels (Keum, 2020). To avoid the risk contagion, other affiliates tend to support the underperforming affiliates by way of resource allocation. Consequently, the business group resources are transferred from the outperforming to the underperforming affiliates (Bardolet et al., 2017; Busenbark et al., 2022).

Taken together, based on the resource demands of the underperforming affiliates and the risk contagion aversion of outperforming affiliates, the principle of resource allocation within the business group follows the co-insurance theory. Hence, more resources are allocated to underperforming affiliates and less resources to outperforming affiliates.

*Hypothesis 2a*. A negative feedback on an affiliate’s performance is positively correlated with resource allocation.

*Hypothesis 2b*. A positive feedback on an affiliate’s performance is negatively correlated with resource allocation.

### The mediating influence of hierarchy management on the relationship between the affiliate performance feedback and technology innovation

The parent firm generally possesses advanced knowledge and information (Nell and Ambos, 2013; Feldman, 2021), which can be transferred to its affiliates through the hierarchy management (Beugelsdijk and Jindra, 2018; Zeng et al., 2018). Considering the managerial involvement is not always efficient (Asakawa et al., 2018; Wang and Wang, 2021), the parent firm can adjust the level of hierarchy management based on the affiliate performance feedback.

When an affiliate’s performance is below its aspired level, both the parent firm and affiliate become motivated to seek potential solutions to reach the aspired level. Technology innovation may allow underperforming affiliates to deal with the performance issues by improving its market competitiveness (Greve, 2003; Saemundsson et al., 2022). However, innovation may be fraught with risks and uncertainties (Li and Vermeulen, 2021; Dean et al., 2022). Underperforming affiliates have a higher risk tolerance, as they do not have much to lose (Lim, 2019). In this scenario, the technology innovation may lead to lower economic returns with a higher variance (Bowman, 1980; Henkel, 2009; Li and Vermeulen, 2021), further exacerbating performance shortfalls. To reduce the innovation risk, the parent firm tends to guide the technology innovation of underperforming affiliates through a stronger hierarchical management. Hence, the hierarchy management as a response to the negative performance feedback of an affiliate can prompt the affiliate’s technology innovation.

When the affiliate’s performance is above its aspired level, both the parent firm and affiliate are satisfied with the returns and have less motivation to pursue an innovation strategy (Cyert and March, 1963; Greve, 2003; Argote and Greve, 2007). In this scenario, the parent firm should reduce the level of hierarchy management to avoid the inefficiency of attention focus (Sengul and Obloj, 2017; Eklund and Mannor, 2021). The reduced hierarchy management makes the operation of the affiliates similar to that of stand-alone firms, and finally, the outperforming affiliates present weak motivation to pursue the technology innovation.

Taken together, the influence of hierarchy management on affiliate’s technology innovation varies with the affiliate performance feedback. Specifically, a strong hierarchical management is convenient for guiding underperforming affiliates’ technology innovation, and a reduced level of hierarchical management leads to less technology innovation for outperforming affiliates.

*Hypothesis 3a*. A higher level of hierarchical management of underperforming affiliates facilitates the affiliate’s technology innovation.

*Hypothesis 3b*. A lower level of hierarchical management of outperforming affiliates impedes the affiliate’s technology innovation.

### The mediating influence of resource allocation on the relationship between the affiliate performance feedback and technology innovation

The business group can provide valuable resources such as knowledge, capital, and information, which are hard to obtain from external markets (Chang et al., 2006; Khanna and Yafeh, 2007; Dou et al., 2021). To ensure that resources are allocated efficiently, the parent firm should take the resource needs of affiliates with different performance feedbacks into account.

When an affiliate’s performance is below its aspired level, both the parent firm and affiliate tend to employ technology innovation to solve the problem (Rhee et al., 2019). However, technology innovation requires a huge amount of resources. The performance shortfall reduces the total resources of affiliates and inhibits the innovation decision (Smulowitz et al., 2020). In this scenario, the parent firm can allocate more resources to support them. Hence, underperforming affiliates can pursue technology innovation in the context that resource constraints are alleviated through the resource allocation within the business group (Belenzon and Berkovitz, 2010; Arrfelt et al., 2013; Tan et al., 2018; Dou et al., 2021).

When an affiliate’s performance is above its aspired level, both the parent firm and affiliate are satisfied with the returns and have less motivation to invest in technology innovation (Greve, 2003). Meanwhile, receiving a positive performance feedback brings more resources to an affiliate. To ensure that these slack resources are efficiently used, the parent firm can transfer these resources to other affiliates that need them (Bardolet et al., 2017). Consequently, the reduced resources constrain the technology innovation of outperforming affiliates.

Taken together, the way resource allocation is carried out has a significant mediating effect on the relationship between the performance feedback and technology innovation of affiliates. Specifically, the parent firm allocates more resources to the underperforming affiliates to support their technology innovation, while less resources are assigned to the outperforming affiliates who have a lower motivation for innovation.

*Hypothesis 4a*. The increased resource allocation prompts the technology innovation of underperforming affiliates.

*Hypothesis 4b*. The decreased resource allocation impedes the technology innovation of outperforming affiliates.

**Figure 1:**
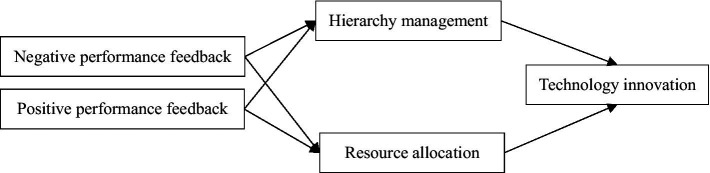
Theoretical framework.

Based on above hypotheses, the conceptual framework of this paper is shown in the Figure 1.

## Methods

### Data and sample

We select a sample of business groups in China, the largest emerging economy, to verify how parent functions affect the relationship between the affiliate performance feedback and technology innovation, given that business groups exist to overcome the inefficiency of external markets (Yiu et al., 2005; Chang et al., 2006; Khanna and Yafeh, 2007; Belenzon and Berkovitz, 2010), and parent functions need to be strengthened to promote the business group’s competitive advantages in emerging economies (Zeng et al., 2018; Meyer et al., 2020). Following La Porta et al. (1999), we define a business group as one in which the parent firm owns more than 10% of the equity of at least five affiliates. The concentration of ownership is convenient for the parent firm to manage and monitor its affiliates, and the internal market, including multiple affiliates, provides a chance to verify the criterion of resource allocation across affiliates within the business group.

We select 2010–2020 data of listed affiliates. Our data are from the *China Stock Market & Accounting Research Database*. The performance level of affiliates is measured by the ROA from year *t-2* to year *t*, such that the 2010 and 2011 data of affiliates are used to measure the variable. To reduce potential endogeneity impacts, the mediating variable is lagged by 1 year, and the dependent variable is lagged by 2 years. Therefore, the former variable covers the 2013–2019 period, and the latter covers 2014–2020. Other variables cover the 2012–2018 period. Moreover, we delete the data of affiliates in the financial industry because this industry’s financial structure differs from others. We also exclude affiliates with missing data or those lagged with ST or *ST (special treatment because of financial and operational problems). To reduce the influence of outliers, we winsorize all continuous variables at 1%.

### Variables

#### Performance feedback

The aspired firm performance level is often based on historical and peer aspirations. The former weighs previous performance and previously aspired levels to form the current aspired level (Cyert and March, 1963; Kuusela et al., 2017). The latter represents a comparison with a reference group of other firms, such as firms in the same industry (Ye et al., 2021). In contrast to peer aspiration, historical aspiration is based on the firm’s previous performance and reflects the firm’s capability (Kim et al., 2015). The peer aspiration depends on the results of peer performance. In this regard, the firm may attribute an outperformance to its own ability and present more confidence in its future actions (Ye et al., 2021), and this ambiguous attribution may engender distortion of its response (Schumacher et al., 2020). By contrast, the performance feedback based on historical aspirations is less ambiguous, and thus can better guide a firm to make an objective response. Hence, we employ the historical aspiration to measure the performance feedback. Historical aspiration is often defined as follows: *A_t_* = 𝛼*P*_*t–*1_ + (1 − 𝛼)*A*_*t–*1_. Referring to Kuusela et al. (2017), we choose a fixed value of 𝛼 (𝛼=0.75) to verify our hypotheses, and we also employ 𝛼=0.6 and 𝛼=0.5 in the robustness tests.

We use spline functions to measure the performance feedback. Based on the knots at the extreme values and zero, the spline function of ROA-historical aspiration creates two separate variables: ROA-historical aspiration >0, and ROA-historical aspiration <0. The former variable (*ROAH*) accounts for positive performance feedback, and the latter (*ROAL*) indicates negative performance feedback.

#### Hierarchy management

Hierarchy management refers to the control degree of the parent firm on group affiliates, which is often exercised through board memberships (Zheng et al., 2022). It is the responsibility of the board to come up with an innovation strategy (Jia et al., 2018), and the parent firm exercises considerable control over its affiliates through their power to appoint affiliate directors (Jia et al., 2018). People working in the parent firm have more talent, experience, and social capital, who, through the affiliates’ boards, can help affiliates search for the problems and corresponding solutions. Hence, following Zheng et al. (2022), we measure the hierarchy management (*Hierarchy*) as the ratio of the number of affiliate directors who are working concurrently in the parent firm to the size of the affiliate’s board.

#### Resource allocation

Compared with stand-alone firms, a business group’s competitive advantage is that its resources can be allocated efficiently across affiliates (Chang et al., 2006; Belenzon and Berkovitz, 2010; Manikandan and Ramachandran, 2015). However, the allocation leads to more resources for some affiliates and less for others. The resource change for an affiliate is measured by subtracting resource outflows from resource inflows during transactions between that affiliate and the parent firm and other affiliates. Finally, the resource allocation (*Allocation*) is measured by the ratio of the resource change to the operating income of affiliates (Jia et al., 2013; Zheng et al., 2022).

#### Technology innovation

There are two methods to measure the technology innovation: research and development (R&D) and innovation patents (Chang et al., 2006; Wang and Wang, 2021). Compared with R&D expenditure, patents can precisely represent the innovation abilities of firms (Zheng et al., 2022), as R&D expenditures do not always successfully convert into innovation patents (Li and Vermeulen, 2021; Dean et al., 2022). Hence, we employ the innovation patent variable to avoid the ambiguous effect of parent functions on the affiliate’s technology innovation. Technology innovation (*Inno*) is measured by the natural logarithm of one plus the number of innovation patents for which the affiliate applied.

#### Control variables

We include several control variables that can affect the affiliate’s technology innovation. In particular, we include variables characterizing the affiliate. Since technology innovation has a significant relationship with R&D expenditure, we include the affiliate’s R&D, measured by the ratio of R&D expenditure to operating income (Zheng et al., 2022). To control for differences between young and old affiliates, we include the affiliate’s age, measured by the natural logarithm of its founding year. To control for differences between small and large affiliates, we include the affiliate’s size, measured by the natural logarithm of the affiliate’s total assets (Vissa et al., 2010). As technology innovation requires a huge amount of resources, and the resources possessed by affiliates affect their technology innovation, we include slack resources (*Slack*), measured by the current assets divided by current liabilities (Chen and Miller, 2007; Arrfelt et al., 2013). We also control for the asset-liability ratio (*Leverage*; measured by the ratio of the total debt to assets), as it affects the affiliate’s financial ability to pursue innovation (Zhang et al., 2022). Bankruptcy risk may impact an affiliate’s innovation because of operational and financial trouble, and the Altman’s (1983) Z-score indicates a firm’s distance from bankruptcy. A lower score shows that a firm is on the verge of bankruptcy (Chen and Miller, 2007).

We also include some variables characterizing the interaction between affiliates and the parent firm. We control for the property attribute (*State*)—if the firm is affiliated to a state-owned business group, we assign it a value of one, and zero otherwise (Zheng et al., 2022). Moreover, the position of an affiliate in the pyramidal ownership structure influences its relationship with the parent firm (Belenzon et al., 2019), which impacts the affiliate’s innovation (Zheng et al., 2022). Hence, we include the control level, measured by one plus the number of firms in the middle between the affiliate and parent firm. Because the equity ratio impacts the degree of the parent firm’s control and management of the affiliate’s technology innovation (Mahmood et al., 2017; Feldman, 2021), we include ownership, measured by the ownership ratio of the parent firm in the affiliate. The degree of the parent firm’s involvement in the affiliate’s technology innovation is affected by other shareholders. We include equity restriction, measured by the ratio of ownership of the parent firm to the ownership that remains after subtracting the parent firm’s ownership from the equity of the top 10 shareholders. In addition, the industry and year in which an affiliate operates may impact its innovation decision; thus, we control for a set of dummy variables for the year and industry.

### Model

To verify our hypotheses, we employ the following regression models:


(1) $$Inno=\alpha_{0}+\alpha_{1}ROAL+\alpha_{2}ROAH+\sum Controls+\varepsilon$$

(2) $$ME=\alpha_{0}+\alpha_{1}ROAL+\alpha_{2}ROAH+\sum Controls+\varepsilon$$

(3) $$Inno=\alpha_{0}+\alpha_{1}ME+\alpha_{2}ROAL+\alpha_{3}ROAH+\sum Controls+\varepsilon$$

For our analyses, first, we verify in Eq. (1) the direct influence of the affiliate performance feedback on technology innovation. Second, in Eq. (2), we examine how the parent firm responds to the affiliate’s performance level. *ME* represents the variables of hierarchy management and resource allocation. Third, we verify in Eq. (3) the mediating effect of parent functions on the relationship between the affiliate’s performance feedback and technology innovation. The three equations follow a sequential order; the previous equation is significant for the next equation. The mediating effect is verified when the influences of the variables in the three equations are all significant.

## Results

### Descriptive statistics and correlation

Table 1 provides the descriptive statistics and correlation for all variables. As the results of the descriptive statistics show, the average of hierarchy management is 0.27, indicating that approximately 27% of affiliate directors are from the parent firm. The average of resource allocation is −0.24, demonstrating that the resource allocation leads to a resource reduction for the listed affiliates in the business group. There are small differences in the average and standard deviation between the negative and positive performance feedbacks of affiliates.

**Table 1:** Descriptive statistics and correlation.

	1	2	3	4	5	6	7	8	9	10	11	12	13	14	15
1.Inno	1.00														
2.Hierarchy	0.05	1.00													
3.Allocation	0.05	−0.01	1.00												
4.RoaL	0.04	0.04	0.05	1.00											
5.RoaH	−0.02	−0.04	−0.04	0.21	1.00										
6.Level	0.01	0.05	−0.00	−0.01	0.04	1.00									
7.State	0.02	0.10	−0.01	0.05	−0.02	0.13	1.00								
8.Ownership	0.01	0.21	0.02	0.07	−0.03	−0.07	0.12	1.00							
9.Restriction	−0.02	0.16	−0.03	0.01	0.01	−0.01	0.18	0.56	1.00						
10.Slack	−0.04	0.03	−0.18	−0.08	0.04	0.02	0.19	0.00	0.07	1.00					
11.Z-score	0.00	−0.06	0.13	−0.03	0.10	0.02	−0.15	−0.07	−0.07	−0.39	1.00				
12.Size	0.07	0.13	−0.03	0.14	−0.12	−0.02	0.22	0.26	0.04	0.25	−0.41	1.00			
13.Leverage	−0.03	0.06	−0.20	−0.04	−0.01	0.04	0.18	0.03	0.09	0.55	−0.59	0.43	1.00		
14.Age	−0.07	−0.01	−0.08	0.01	0.11	0.20	0.27	−0.20	0.07	0.17	−0.06	0.13	0.22	1.00	
15.R&D	0.12	−0.03	0.09	−0.06	−0.03	0.03	−0.09	−0.08	−0.09	−0.14	0.05	−0.05	−0.13	−0.04	1.00
Mean	0.58	0.27	−0.24	−0.02	0.01	0.92	0.63	0.38	3.72	0.86	4.23	22.58	0.49	2.56	3.84
Std	1.51	0.16	0.66	0.03	0.03	0.30	0.48	0.15	4.38	0.62	5.21	1.33	0.21	0.59	6.77
Min	0.00	0.00	−3.29	−0.21	0.00	0.00	0.00	0.10	0.24	0.06	−0.11	19.54	0.05	0.00	0.00
Max	9.52	0.67	0.67	0.00	0.19	1.79	1.00	0.75	22.32	3.58	36.63	26.21	0.94	3.30	37.93

*N* = 9,480. Correlations with an absolute value of greater than 0.03 are significant at *p* < 0.01.

As the correlation of variables show, parent functions, including hierarchy management and resource allocation, have a significant association with the affiliate performance feedback and technology innovation, which provides an opportunity to verify further the mediating influence of parent functions.

### Hypotheses testing

Table 2 presents the empirical results of the mediating effect of parent functions on the relationship between the affiliate performance feedback and technology innovation. Model 1 is the baseline model; it includes all the control variables. Model 2 examines the direct effect of the affiliate performance feedback on technology innovation. Model 3 verifies how the parent responds, through its level of hierarchical management, to the affiliate’s performance level. Model 4 tests the influence of the affiliate performance feedback on the resource allocation within the business group. Model 5 investigates the mediating effect of the parent’s hierarchy management on the relationship between the affiliate performance feedback and technology innovation. Model 6 explores the mediating effect of resource allocation on the relationship between the affiliate performance feedback and technology innovation.

**Table 2:** Results of the mediating effect of the parent’ response.

	Model 1	Model 2	Model 3	Model 4	Model 5	Model 6
	Inno	Inno	Hierarchy	Inno	Allocation	Inno
RoaL		1.663^***^	0.125^**^	1.635^***^	1.038^***^	1.610^***^
		(3.78)	(2.43)	(3.71)	(4.00)	(3.66)
RoaH		−0.965^**^	−0.204^***^	−0.918^*^	−0.547^*^	−0.938^*^
		(−2.01)	(−3.71)	(−1.91)	(−1.70)	(−1.96)
Hierarchy				0.230^**^		
				(2.23)		
Allocation						0.051^**^
						(2.56)
Level	0.033	0.035	0.031^***^	0.028	0.015	0.035
	(0.65)	(0.70)	(5.42)	(0.55)	(0.68)	(0.68)
State	0.191^***^	0.186^***^	0.014^***^	0.183^***^	0.004	0.186^***^
	(5.78)	(5.64)	(3.80)	(5.53)	(0.31)	(5.64)
Equity	−0.238^*^	−0.238^*^	0.165^***^	−0.276^*^	0.024	−0.239^*^
	(−1.70)	(−1.70)	(11.08)	(−1.95)	(0.42)	(−1.71)
Balance	0.002	0.002	0.002^***^	0.001	−0.004^*^	0.002
	(0.41)	(0.39)	(4.71)	(0.26)	(−1.88)	(0.43)
Slack	−0.068^***^	−0.057^**^	−0.005	−0.056^**^	−0.152^***^	−0.049^*^
	(−2.66)	(−2.21)	(−1.42)	(−2.17)	(−7.55)	(−1.91)
Z-score	0.004	0.005	−0.000	0.005	0.004^***^	0.004
	(1.07)	(1.38)	(−0.07)	(1.39)	(2.73)	(1.32)
Size	0.148^***^	0.138^***^	0.010^***^	0.135^***^	0.043^***^	0.135^***^
	(9.29)	(8.44)	(6.51)	(8.24)	(6.68)	(8.28)
Lev	−0.125	−0.082	0.029^**^	−0.089	−0.335^***^	−0.065
	(−1.29)	(−0.84)	(2.31)	(−0.91)	(−5.80)	(−0.66)
Age	−0.141^***^	−0.137^***^	0.001	−0.137^***^	0.013	−0.137^***^
	(−4.87)	(−4.66)	(0.31)	(−4.67)	(1.14)	(−4.69)
R&D	0.012^***^	0.012^***^	−0.001^***^	0.012^***^	0.000	0.012^***^
	(4.03)	(4.12)	(−3.37)	(4.19)	(0.11)	(4.11)
Year	Yes	Yes	Yes	Yes	Yes	Yes
Industry	Yes	Yes	Yes	Yes	Yes	Yes
_cons	−2.808^***^	−2.571^***^	−0.072^*^	−2.554^***^	−1.046^***^	−2.517^***^
	(−8.21)	(−7.34)	(−1.93)	(−7.28)	(−6.98)	(−7.17)
N	9,480	9,480	9,480	9,480	9,480	9,480
F	36.91	34.75	22.14	33.91	22.63	33.72
R-sq	0.084	0.086	0.075	0.086	0.156	0.086

* *p* < 0.1 (significance level).

** *p* < 0.05 (significance level).

*** *p* < 0.01 (significance level).

The empirical results of the control variables in Model 1 show that R&D expenditure and size have a positive correlation with the affiliate’s technology innovation; equity and slack have an adverse effect on the affiliate’s technology innovation, and state-owned affiliates have a higher innovation capability compared with private-owned ones. The level of significance indicates that the chosen control variables are relatively effective.

Based on the BTOF, the performance feedback has a significant impact on technology innovation (Greve, 2003; Chen and Miller, 2007; Bromiley and Washburn, 2011; Ye et al., 2021). The results of Model 2 support this theory. Specifically, the coefficient of a negative performance feedback is positive and significant (*α* = 1.663, *p* < 0.01), while the coefficient of a positive performance feedback is negative and significant (*α* = −0.965, *p* < 0.05), demonstrating that receiving a negative performance feedback facilitates the affiliate’s technology innovation, while receiving a positive performance feedback inhibits it. Model 2 shows that the technology innovation varies with the performance feedback.

Hypothesis 1a predicts that the parent firm will strengthen its hierarchical management on underperforming affiliates. In line with this hypothesis, the coefficient of a negative performance feedback in Model 3 is significant and positive (*α* = 0.125, *p* < 0.05). Hypothesis 1b proposes that the parent firm will reduce its degree of hierarchical management of outperforming affiliates. The empirical result shows that the coefficient of a positive performance feedback is significant and negative (*α* = −0.204, *p* < 0.01), supporting our conjecture. Model 3 shows that the parent firm should consider the affiliates’ performance level when adjusting its hierarchical management of these affiliates.

Hypothesis 2a proposes that a business group’s resources are allocated to support the underperforming affiliates. The empirical results of Model 4 show that the coefficient of a negative performance feedback is significant and positive (*α* = 1.038, *p* < 0.01). Hypothesis 2b predicts that the resource allocation will reduce the amount of resources possessed by the outperforming affiliates. Consistent with the hypothesis, the coefficient of a positive performance feedback is significant and negative (*α* = −0.547, *p* < 0.1). Model 4 shows that the parent firm allocates resources across affiliates following the co-insurance theory; the parent firm allocates more resources to underperforming affiliates and less resources to outperforming ones.

Hypothesis 3a predicts that a stronger hierarchical management of an underperforming affiliate facilitates its technology innovation. Integrating models 2 and 3, the empirical results of model 4 show that the coefficient of hierarchy management is significant and positive (*α* = 0.230, *p* < 0.05); the coefficient of a negative performance feedback is significant and positive (*α* = 1.635, *p* < 0.01), but it is lower than the coefficient in model 2 (1.635 < 1.663), thus supporting our hypothesis. Hypothesis 3b predicts that the reduced hierarchy management will impede the outperforming affiliate’s technology innovation. As model 4 shows, the coefficient of a positive performance feedback is significant and negative (*α* = −0.918, *p* < 0.1), and the absolute value of the coefficient is lower compared with the coefficient in model 2 (0.918 < 0.965). Hypothesis 3b is thus verified. However, the mediating effect of hierarchy management is notably weak and unstable when the affiliate’s performance is above its aspired level. The results of model 4 suggest that affiliates, especially the underperforming ones, should consider taking advantage of the parent firm’s hierarchical management when employing technology innovation to respond to a negative performance feedback.

Hypothesis 4a predicts that the increased resource allocation will prompt the technology innovation of underperforming affiliates. Integrating models 2 and 3, the empirical results of model 4 show that the coefficient of resource allocation is significant and positive (*α* = 0.051, *p* < 0.05); the coefficient of a negative performance feedback is significant and positive (*α* = 1.610, *p* < 0.01), but it is lower than the coefficient in model 2 (1.610 < 1.663), thus supporting our conjecture. Hypothesis 4b predicts that the decreased resource allocation will inhibit the technology innovation of outperforming affiliates. The coefficient of an affiliate’s positive performance feedback is significant and negative (*α* = −0.938, *p* < 0.1), and the absolute value of the coefficient is lower compared with the coefficient in model 2 (0.938 < 0.965). Hypothesis 4b is thus verified. The results of model 6 show that the resource allocation diversely impacts the technology innovation of affiliates who have different performance levels.

### Robustness tests

We subject the results to several robustness tests. First, we employ a bootstrapping approach to test for the mediating effect of parent functions (Hayes, 2017). Based on bootstrapping procedures with 500 resamples, the results in Table 3 show that the indirect effect of an affiliate’s negative performance feedback on its technology innovation through parent functions (hierarchy management and resource allocation) is significant. Moreover, the bias-corrected 95% confidence interval (CI) does not contain zero. Thus, hypotheses 3a and 4a are supported.

**Table 3:** Results of the bootstrap test for the mediating effect of the parent’s response.

Total and specific indirect effect	Coeff	SE	Bootstrapping BC 95% CI	
			Lower	Upper
Negative performance feedback-technology innovation of affiliation				
Sum of indirect effect	1.663	0.483	0.716	2.611
Specific indirect effect				
ROAL ➔ Hierarchy ➔ Inno	1.635	0.458	0.778	2.531
ROAL ➔ Allocation ➔ Inno	1.61	0.424	0.754	2.395
Positive performance feedback-technology innovation of affiliation				
Sum of indirect effect	−0.965	0.551	−2.046	0.115
Specific indirect effect				
ROAH ➔ Hierarchy ➔ Inno	−0.918	0.502	−1.866	0.049
ROAH ➔ Allocation ➔ Inno	−0.938	0.504	−1.89	0.102

Second, we verify the nonlinear effect of the performance feedback on technology innovation. We add a new variable, which is the remainder after subtracting the ROA from the historical aspiration (*HROA*), and the square of the variable (*HROA^2^*) to our models. As Table 4 shows, the closer the performance is to the aspired level, the more motivated an affiliate is to engage in technology innovation; the intensity of the parent’s hierarchical management and resource allocation exert mediating effects on the U-shaped relationship between the affiliate performance feedback and technology innovation. The empirical results of Table 4 support the conjecture that the technology innovation varies with the performance feedback.

**Table 4:** Results of the nonlinear effect of the performance feedback on technology innovation.

	Model 1	Model 2	Model 3	Model 4	Model 5
	Inno	Hierarchy	Inno	Allocation	Inno
Hierarchy			0.235^**^		
			(2.28)		
Allocation					0.053^***^
					(2.64)
HROA^2^	−4.432^**^	−1.096^***^	−4.175^*^	−4.760^***^	−4.181^*^
	(−2.02)	(−4.47)	(−1.90)	(−3.18)	(−1.91)
HROA	0.390	−0.049	0.401	0.225	0.378
	(1.41)	(−1.49)	(1.45)	(1.21)	(1.36)
Level	0.035	0.031^***^	0.027	0.016	0.034
	(0.68)	(5.45)	(0.53)	(0.71)	(0.66)
State	0.188^***^	0.014^***^	0.185^***^	0.004	0.188^***^
	(5.70)	(3.78)	(5.58)	(0.30)	(5.69)
Equity	−0.243^*^	0.165^***^	−0.282^**^	0.022	−0.244^*^
	(−1.74)	(11.06)	(−1.99)	(0.39)	(−1.75)
Balance	0.002	0.002^***^	0.001	−0.004^*^	0.002
	(0.39)	(4.68)	(0.26)	(−1.91)	(0.44)
Slack	−0.063^**^	−0.005	−0.062^**^	−0.153^***^	−0.055^**^
	(−2.43)	(−1.46)	(−2.38)	(−7.62)	(−2.12)
Z-score	0.004	0.000	0.004	0.004^***^	0.004
	(1.24)	(0.00)	(1.25)	(2.74)	(1.18)
Size	0.142^***^	0.010^***^	0.140^***^	0.043^***^	0.140^***^
	(8.75)	(6.54)	(8.54)	(6.76)	(8.59)
Lev	−0.095	0.030**	−0.102	−0.330^***^	−0.077
	(−0.96)	(2.44)	(−1.03)	(−5.71)	(−0.78)
Age	−0.140^***^	0.001	−0.140^***^	0.013	−0.141^***^
	(−4.78)	(0.35)	(−4.79)	(1.15)	(−4.81)
R&D	0.012^***^	−0.001^***^	0.012^***^	0.000	0.012^***^
	(4.12)	(−3.32)	(4.19)	(0.16)	(4.12)
Year	Yes	Yes	Yes	Yes	Yes
Industry	Yes	Yes	Yes	Yes	Yes
_cons	−2.690^***^	−0.075^**^	−2.672^***^	−1.072^***^	−2.633^***^
	(−7.73)	(−2.04)	(−7.66)	(−7.20)	(−7.55)
N	9,480	9,480	9,480	9,480	9,480
F	34.87	22.30	34.02	22.61	33.80
R-sq	0.085	0.075	0.085	0.156	0.085

* *p* < 0.1 (significance level).

** *p* < 0.05 (significance level).

*** *p* < 0.01 (significance level).

Third, we verify our results using an alternative aspiration level. The referents of aspiration levels often consist of historical and peer performance. Peer aspiration refers to the average of peer performance (Ye et al., 2021). The performance feedback is measured by the comparison between that of an affiliate and its peer. The empirical results (in Table 5) support the mediating effects of parent functions (hierarchy management and resource allocation) on the relationship between an affiliate’s negative performance feedback and its technology innovation. However, the affiliate’s positive performance feedback promotes technology innovation, and the relationship with the parent firm’s response is not significant. The difference between historical and peer aspirations is mainly reflected in the affiliate’s positive performance feedback.

**Table 5:** Results of the tests for the effect of peer performance feedback.

	Model 1	Model 2	Model 3	Model 4	Model 5
	Inno	Hierarchy	Inno	Allocation	Inno
RoaL	0.828^**^	0.165^***^	0.789^*^	1.528^***^	0.752^*^
	(2.03)	(3.44)	(1.93)	(5.96)	(1.84)
RoaH	1.643^***^	−0.032	1.650^***^	0.212	1.632^***^
	(2.84)	(−0.60)	(2.86)	(1.07)	(2.82)
Hierarchy			0.234^**^		
			(2.28)		
Allocation					0.050^**^
					(2.49)
Level	0.034	0.031^***^	0.026	0.016	0.033
	(0.66)	(5.40)	(0.52)	(0.74)	(0.65)
State	0.201^***^	0.015^***^	0.197^***^	0.013	0.200^***^
	(6.12)	(4.06)	(6.00)	(0.93)	(6.10)
Equity	−0.310^**^	0.162^***^	−0.348^**^	−0.013	−0.309^**^
	(−2.19)	(10.77)	(−2.42)	(−0.23)	(−2.19)
Balance	0.004	0.002^***^	0.003	−0.003	0.004
	(0.85)	(4.85)	(0.72)	(−1.33)	(0.88)
Slack	−0.058^**^	−0.005	−0.057^**^	−0.144^***^	−0.051^**^
	(−2.24)	(−1.31)	(−2.20)	(−7.08)	(−1.96)
Z-score	0.001	−0.000	0.001	0.003^**^	0.001
	(0.38)	(−0.28)	(0.39)	(2.23)	(0.33)
Size	0.134^***^	0.011^***^	0.132^***^	0.037^***^	0.132^***^
	(8.22)	(6.59)	(8.00)	(5.58)	(8.08)
Lev	−0.022	0.032^**^	−0.029	−0.272^***^	−0.008
	(−0.22)	(2.50)	(−0.29)	(−4.66)	(−0.08)
Age	−0.146^***^	0.000	−0.146^***^	0.011	−0.146^***^
	(−4.99)	(0.01)	(−4.99)	(0.97)	(−5.01)
R&D	0.012^***^	−0.001^***^	0.013^***^	0.000	0.012^***^
	(4.17)	(−3.23)	(4.24)	(0.37)	(4.16)
Year	Yes	Yes	Yes	Yes	Yes
Industry	Yes	Yes	Yes	Yes	Yes
_cons	−2.563^***^	−0.077^**^	−2.545^***^	−0.956^***^	−2.516^***^
	(−7.33)	(−2.07)	(−7.25)	(−6.26)	(−7.17)
N	9,480	9,480	9,480	9,480	9,480
F	34.29	22.14	33.45	23.78	33.34
R-sq	0.086	0.075	0.087	0.160	0.086

* *p* < 0.1 (significance level).

** *p* < 0.05 (significance level).

*** *p* < 0.01 (significance level).

Fourth, we verify our results using alternative hierarchy management and resource allocation variables. As the parent firm can intervene in the appointment of the affiliate’s executives to ensure the implementation of technology innovation, we measure the hierarchy management variable as the ratio of the number of affiliate directors and executives who are working concurrently in the parent firm to the size of the affiliate’s board and executive group. Capital allocation is the core of resource allocation (Chang et al., 2006; Arrfelt et al., 2015); affiliates can reduce their bankruptcy risk through co-insurance (Jia et al., 2013). Here, capital allocation is measured by the ratio of capital change to income. The empirical results (in Table 6) support the mediating effect of resource allocation after substituting the variables. We surmise that the intervention of affiliate executives may overly restrict the flexibility of affiliates, which has an adverse impact on technology innovation.

**Table 6:** Results of the tests for the alternative variables of parent functions.

	Model 1	Model 2	Model 3	Model 4	Model 5
	Inno	Hierarchy	Inno	Allocation	Inno
RoaL	1.663^***^	0.117^***^	1.641^***^	0.946^***^	1.614^***^
	(3.78)	(2.88)	(3.73)	(3.98)	(3.66)
RoaH	−0.965^**^	−0.174^***^	−0.933^*^	−0.534^*^	−0.938^*^
	(−2.01)	(−4.03)	(−1.94)	(−1.88)	(−1.95)
Hierarchy			0.189		
			(1.51)		
Allocation					0.052^**^
					(2.49)
Level	0.035	0.009^*^	0.034	0.004	0.035
	(0.70)	(1.91)	(0.66)	(0.20)	(0.69)
State	0.186^***^	−0.018^***^	0.189^***^	−0.002	0.186^***^
	(5.64)	(−5.89)	(5.77)	(−0.17)	(5.65)
Equity	−0.238^*^	0.136^***^	−0.264^*^	−0.023	−0.237^*^
	(−1.70)	(11.40)	(−1.87)	(−0.45)	(−1.70)
Balance	0.002	0.001^***^	0.001	−0.001	0.002
	(0.39)	(2.64)	(0.34)	(−0.63)	(0.40)
Slack	−0.057^**^	0.001	−0.057^**^	−0.114^***^	−0.051^**^
	(−2.21)	(0.42)	(−2.22)	(−6.24)	(−1.98)
Z-score	0.005	−0.000	0.005	0.004^***^	0.004
	(1.38)	(−0.02)	(1.39)	(3.04)	(1.32)
Size	0.138^***^	0.008^***^	0.136^***^	0.052^***^	0.135^***^
	(8.44)	(5.87)	(8.29)	(9.09)	(8.25)
Lev	−0.082	0.014	−0.085	−0.417^***^	−0.061
	(−0.84)	(1.38)	(−0.87)	(−7.85)	(−0.61)
Age	−0.137^***^	−0.002	−0.136^***^	0.006	−0.137^***^
	(−4.66)	(−0.80)	(−4.65)	(0.56)	(−4.67)
R&D	0.012^***^	−0.001^***^	0.012^***^	−0.001	0.012^***^
	(4.12)	(−4.12)	(4.17)	(−1.11)	(4.13)
Year	Yes	Yes	Yes	Yes	Yes
Industry	Yes	Yes	Yes	Yes	Yes
_cons	−2.571^***^	−0.035	−2.564^***^	−1.199^***^	−2.508^***^
	(−7.34)	(−1.16)	(−7.31)	(−8.91)	(−7.14)
N	9,480	9,480	9,480	9,480	9,480
F	34.75	17.78	33.88	23.58	33.74
R-sq	0.086	0.057	0.086	0.166	0.086

* *p* < 0.1 (significance level).

** *p* < 0.05 (significance level).

*** *p* < 0.01 (significance level).

Moreover, we employ 𝛼=0.6, 𝛼=0.5 to measure the historical aspiration. The empirical results show that the mediating effect of parent functions is more robust when the affiliate’s performance is below the aspired level.

## Conclusion

### Discussions

How a parent firm affects the affiliate’s technology innovation is crucial to the understanding of parent functions. Using 2010–2020 data of listed affiliates belonging to Chinese business groups, we propose that a negative performance feedback facilitates an affiliate’s technology innovation, while a positive performance feedback inhibits it. Because an affiliate’s performance feedback and technology innovation impact the business group’s welfare, the parent firm tends to respond accordingly through its functions. We focus on the following parent functions: hierarchy management and resource allocation, and find that, the parent firm strengthens its hierarchical management and allocates more resources to the underperforming affiliates to support their technology innovation and reduces its hierarchical management and resource allocation to outperforming affiliates that are reluctant to engage in technology innovation. Hence, the technology innovation of an underperforming affiliate is subject to parent functions more so than that of outperforming affiliates.

### Practical implications

We investigate the mediating effect of parent functions on the relationship between the affiliate performance feedback and technology innovation. Some practical implications for the affiliates and parent firms are provided.

First, we propose that whether affiliates engage in technology innovation depends on its performance feedback. When the performance achieves the aspired level, the affiliate is less motivated to engage in technology innovation. Otherwise, the affiliate can reach the aspired level through technology innovation. Our results are consistent with the view of most research on the BTOF (Greve, 2003; Choi et al., 2019; Cheng et al., 2022).

Second, we recommend the parent firm to adjust the intensity of its hierarchical management on its affiliates based on the affiliate performance feedback. Recent literature mainly focuses on the impact of board (Choi et al., 2019; Cheng et al., 2022) and top managers (Schumacher et al., 2020; Saemundsson et al., 2022) on the relationship between firm underperforming and risky decisions, devoting limited attention to the influence of the controlling shareholders (Vissa et al., 2010; Joseph et al., 2016; Rhee et al., 2019). Compared to stand-alone firms, affiliated firms are managed by their parent firms. Considering the unclear relationship between parent management and affiliate development (Raab et al., 2014; Palmié et al., 2016; Asakawa et al., 2018; Beugelsdijk and Jindra, 2018; Wang and Wang, 2021), we recommend the parent firm to strengthen its hierarchical management on underperforming affiliates in a way of people who are from the parent firm serving in the affiliate board, and reduce that on outperforming affiliates, based on the performance level of affiliates.

Third, we provide some reference on the resource allocation within a business group. A business group is more than a simple sum of affiliates given the interactions among them (Rhee et al., 2019; Keum, 2020). The affiliates’ competitive advantages lie in the resource transferring and sharing among firms within the business group (Hu et al., 2019; Sengul et al., 2019). Considering the debate on the winner-picking and co-insurance theories (Jia et al., 2013; Busenbark et al., 2022), we propose that the resource allocation should consider the innovation motivation and slack resources of affiliates. Drawing on the BTOF, our study supports the co-insurance theory. Specifically, the parent firm can transfer resources from outperforming affiliates to underperforming affiliates to support their technology innovation.

### Limitations and avenues for future research

In this study, we examine how the parent firm responds to the affiliate’s performance feedback and how the response influences the affiliate’s technology innovation, thus providing a view of parent functions. However, this study has several limitations, which provide directions for future research.

First, affiliates simultaneously operate within the business group and external markets (Meyer et al., 2020). Some factors from the external markets also impact the affiliate’s technology innovation (Fan et al., 2020). Given that the business group is treated as a substitute for an inefficient external market, future studies can investigate the influence of parent functions on the relationship between an affiliate’s performance feedback and its technology innovation under different external market contexts.

Second, we recommend the parent firm to adjust the intensity of its hierarchical management to respond to the affiliate’s performance feedback, but the adjustment is largely affected by the business group’s ownership structure. Future studies can address the question of to what extent should a parent firm adjust its hierarchical management that in a way that is considered reasonable within its ownership scope.

Third, we find that the allocation of resources leads to reduced resources for outperforming affiliates, which harms the interest of other shareholders (Fan et al., 2016). Future studies can explore how other shareholders respond to the resource allocation of the parent firm.

## Data availability statement

The original contributions presented in the study are included in the article/supplementary material, further inquiries can be directed to the corresponding authors.

## Author contributions

LZ and BS developed the idea, contributed to and wrote the manuscript, and approved the submitted version.

## Funding

This paper is financially supported by the Natural Science Foundation of Shandong Province (ZR2020QG012).

## Conflict of interest

The authors declare that the research was conducted in the absence of any commercial or financial relationships that could be construed as a potential conflict of interest.

## Publisher’s note

All claims expressed in this article are solely those of the authors and do not necessarily represent those of their affiliated organizations, or those of the publisher, the editors and the reviewers. Any product that may be evaluated in this article, or claim that may be made by its manufacturer, is not guaranteed or endorsed by the publisher.
